# Target-Driven Tissue-Agnostic Drug Approvals—A New Path of Drug Development

**DOI:** 10.3390/cancers16142529

**Published:** 2024-07-13

**Authors:** Kyaw Z. Thein, Yin M. Myat, Byung S. Park, Kalpana Panigrahi, Shivaani Kummar

**Affiliations:** 1 Division of Hematology and Medical Oncology, Comprehensive Cancer Centers of Nevada—Central Valley, 3730 S Eastern Ave, Las Vegas, NV 89169, USA; 2 Department of Medicine, Kirk Kerkorian School of Medicine, University of Nevada Las Vegas (UNLV), 4505 S, Maryland Pkwy, Las Vegas, NV 89154, USA; 3 College of Osteopathic Medicine, Touro University Nevada, Touro College and University System, 874 American Pacific Dr, Henderson, NV 89014, USA; 4 Belfield Campus, University College Dublin (UCD) School of Medicine, D04 V1W8 Dublin, Ireland; ym.myat8@gmail.com; 5 Department of Internal Medicine, One Brooklyn Health—Interfaith Medical Center Campus, 1545, Atlantic Avenue, Brooklyn, NY 11213, USA; kpanigrahi@interfaithmedical.org; 6 OHSU-PSU School of Public Health, Portland, OR 97201, USA; parkb@ohsu.edu; 7 Biostatistics Shared Resource, OHSU Knight Cancer Institute, OHSU School of Medicine, Portland, OR 97239, USA; 8 Division of Hematology & Medical Oncology, Center for Experimental Therapeutics, Knight Cancer Institute, Oregon Health & Science University, 3181 SW Sam Jackson Park Rd., Portland, OR 97239, USA; kummar@ohsu.edu

**Keywords:** tissue-agnostic drug development, pembrolizumab, microsatellite instability-high (*MSI-H*), tumor mutational burden-high (*TMB-H*), dostarlimab-gxly, larotrectinib, entrectinib, neurotrophic tyrosine kinase (*NTRK*), dabrafenib and trametinib, V-Raf murine sarcoma viral oncogene homolog B *V600E* (*BRAF^V600E^*), selpercatinib, rearranged during transfection (*RET*), precision oncology

## Abstract

**Simple Summary:**

Precision oncology is at the forefront of personalized cancer care, utilizing tumor-agnostic therapies that target specific biomarkers, such as tumor mutational burden, microsatellite instability, mutations, or fusions, regardless of cancer type. This review aims to provide a comprehensive summary of the immune checkpoint inhibitors and the targeted therapies approved by the US Food and Drug Administration (FDA) for tumor-agnostic use. We thoroughly reviewed pembrolizumab, dostarlimab, larotrectinib, entrectinib, selpercatinib, dabrafenib plus trametinib, and trastuzumab deruxtecan across eight indications, detailing their efficacy, toxicity, and regulatory history. Additionally, we highlight the critical role of biostatistics in designing the clinical trials necessary for evaluating these treatments. These insights advance our understanding of biomarker-driven approaches, guide future research directions, and support the development of more effective, personalized cancer treatments across diverse histology.

**Abstract:**

The regulatory approvals of tumor-agnostic therapies have led to the re-evaluation of the drug development process. The conventional models of drug development are histology-based. On the other hand, the tumor-agnostic drug development of a new drug (or combination) focuses on targeting a common genomic biomarker in multiple cancers, regardless of histology. The basket-like clinical trials with multiple cohorts allow clinicians to evaluate pan-cancer efficacy and toxicity. There are currently eight tumor agnostic approvals granted by the Food and Drug Administration (FDA). This includes two immune checkpoint inhibitors, and five targeted therapy agents. Pembrolizumab is an anti-programmed cell death protein-1 (PD-1) antibody that was the first FDA-approved tumor-agnostic treatment for unresectable or metastatic microsatellite instability-high (*MSI-H*) or deficient mismatch repair (*dMMR*) solid tumors in 2017. It was later approved for tumor mutational burden-high (*TMB-H*) solid tumors, although the TMB cut-off used is still debated. Subsequently, in 2021, another anti-PD-1 antibody, dostarlimab, was also approved for *dMMR* solid tumors in the refractory setting. Patients with fusion-positive cancers are typically difficult to treat due to their rare prevalence and distribution. Gene rearrangements or fusions are present in a variety of tumors. Neurotrophic tyrosine kinase (*NTRK*) fusions are present in a range of pediatric and adult solid tumors in varying frequency. Larotrectinib and entrectinib were approved for neurotrophic tyrosine kinase (*NTRK*) fusion-positive cancers. Similarly, selpercatinib was approved for rearranged during transfection (*RET*) fusion-positive solid tumors. The FDA approved the first combination therapy of dabrafenib, a B-Raf proto-oncogene serine/threonine kinase (BRAF) inhibitor, plus trametinib, a mitogen-activated protein kinase (MEK) inhibitor for patients 6 months or older with unresectable or metastatic tumors (except colorectal cancer) carrying a *BRAF^V600E^* mutation. The most recent FDA tumor-agnostic approval is of fam-trastuzumab deruxtecan-nxki (T-Dxd) for HER2-positive solid tumors. It is important to identify and expeditiously develop drugs that have the potential to provide clinical benefit across tumor types.

## 1. Introduction

**Figure 1:**
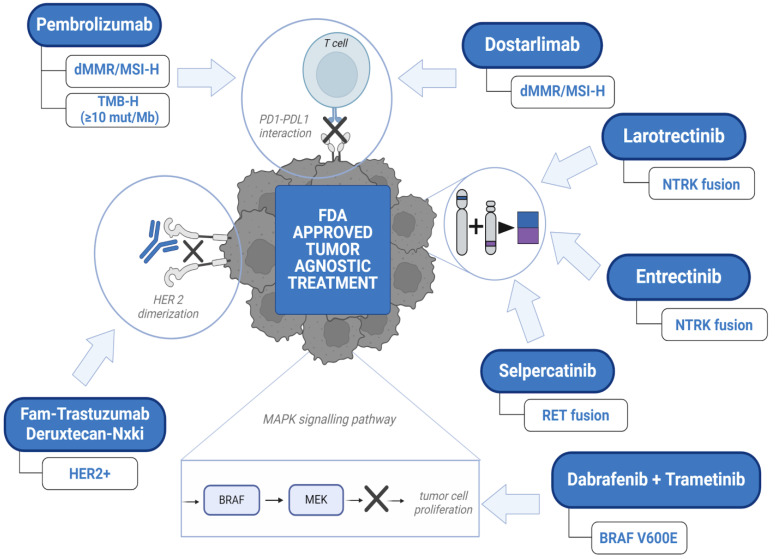
Overview of the FDA-approved tumor-agnostic treatment. Abbreviations: Microsatellite instability-high (MSI-H); mutations/megabase (mut/Mb); deficient mismatch repair (dMMR); programmed cell death protein-1 (PD-1); programmed cell death ligand-1 (PD-L1); tumor mutational burden-high (TMB-H); B-Raf pro-to-oncogene serine/threonine kinase (*BRAF*); human epidermal growth factor receptor 2 (HER2); mitogen-activated protein kinase (*MEK*); neurotrophic tyrosine receptor kinase (*NTRK*); rearranged during transfection (*RET*). X denotes the site of the action of the drug via their mechanism of action.

The regulatory approvals of tumor-agnostic therapies have led to the re-evaluation of the drug development process. Conventional models of drug development are histology-based. On the other hand, the tumor-agnostic drug development of a new drug (or combination) focuses on targeting a common genomic biomarker in multiple cancers, regardless of histology [1]. Basket-like clinical trials with multiple cohorts allow clinicians to evaluate pan-cancer efficacy and toxicity. There are currently eight tumor-agnostic approvals granted by the FDA (Figure 1). This includes two immune checkpoint inhibitors, and five targeted therapy agents. Pembrolizumab is an anti-programmed cell death protein-1 (PD-1) antibody that was the first FDA-approved tumor-agnostic treatment for unresectable or metastatic microsatellite instability-high (*MSI-H*) or deficient mismatch repair (*dMMR*) solid tumors in 2017. It was later approved for tumor mutational burden-high (*TMB-H*) solid tumors, although the *TMB* cut-off used is still debated. Subsequently, in 2021, another anti-PD-1 antibody, dostarlimab, was also approved for *dMMR* solid tumors in the refractory setting. Patients with fusion-positive cancers are typically difficult to treat due to their rare prevalence and distribution. Gene rearrangements or fusions are present in a variety of tumors. Neurotrophic tyrosine kinase (*NTRK*) fusions are present in a range of pediatric and adult solid tumors in varying frequency. Larotrectinib and entrectinib were approved for neurotrophic tyrosine kinase (*NTRK*) fusion-positive cancers. Similarly, selpercatinib was approved for rearranged during transfection (*RET*) fusion-positive solid tumors. The FDA approved the first combination therapy of dabrafenib, a B-Raf proto-oncogene serine/threonine kinase (BRAF) inhibitor, plus trametinib, a mitogen-activated protein kinase (MEK) inhibitor for patients 6 months or older with unresectable or metastatic tumors (except colorectal cancer) carrying a *BRAF^V600E^* mutation. The most recent FDA tumor-agnostic approval is of fam-trastuzumab deruxtecan-nxki (T-Dxd) for HER2-positive solid tumors. It is important to identify and expeditiously develop drugs that have the potential to provide clinical benefits across tumor types.

## 2. Approved Immunotherapy (Checkpoint Inhibitors) in the Field of Tissue-Agnostic Drug Development

### 2.1. Pembrolizumab Approved for Patients with Unresectable or Metastatic dMMR/MSI-H Cancers (23 May 2017)

The MMR system is responsible for detecting and repairing errors made during DNA replication. Mismatch repair deficiency results in a high mutational rate, which contributes to microsatellite instability. The *MMR* genes include mutL homolog 1 (*MLH1*), mutL homolog 2 (*MSH2*), mutL homolog 6 (*MSH6*), and post-meiotic segregation increased 2 (*PMS2*) [2]. The underlying causes of microsatellite instability-high (*MSI-H*) or deficient mismatch repair (*dMMR*) solid tumors are often attributed to inactivated *MMR* genes. This may be due to the methylation of the promoter region or epigenetic changes which may manifest as mutational alterations. This process is also predominant in hereditary cancers such as Lynch syndrome (autosomal dominant) or constitutional mismatch repair deficiency (autosomal recessive).

*MSI-H*/*dMMR* solid tumors may also express PD-1 biomarkers. In a study of 12,019 cancers, 75% exhibited *MMR* deficiency, including adenocarcinomas of the reproductive and gastrointestinal system. Among 11 tumor types, 10% of *dMMR* tumors were early stage, while 5% were late stage, amounting to approximately 60,000 diagnoses annually in the United States [3]. Programmed cell death ligand-1 (PD-L1) blockade increased the levels of functional mutation-associated neoantigens (*MANAs*), suggesting that *dMMR* cancers with high levels of neoantigens are more susceptible to immunotherapy [4]. Pembrolizumab is a monoclonal antibody targeting the PD-1 receptor present on the surface of T cells. Inhibition blocks the interaction with PD-L1/2 ligands, resulting in immunosuppression. This has broad implications for immune surveillance and DNA repair via the mismatch repair system.

**Figure 2:**
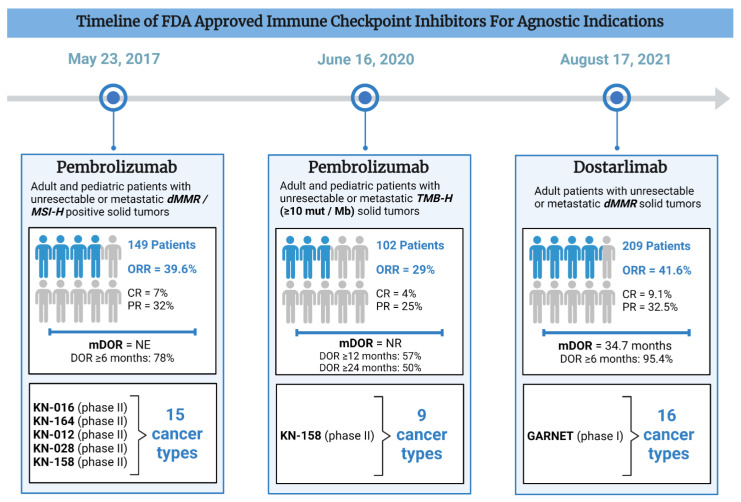
Timeline of FDA-approved immune checkpoint inhibitors for tissue-agnostic indications.

Pembrolizumab was the first tissue-agnostic treatment that received accelerated FDA approval for advanced or metastatic *dMMR*/*MSI-H* solid tumors on 23 May 2017 (Table 1). The efficacy data were based on the five phase II KEYNOTE (KN) trials: KN-016, -164, -012, -028, and -158 (Figure 2). A pooled cohort of 149 patients were studied across 15 different cancer types, including 90 colorectal cancer (CRC) and 59 non-CRC cohorts (FDA, 2017 [5]). The overall objective response rate (ORR) was 39.6% (95% CI 31.7–47.9). In CRC, the ORR was 36%, whereas all other cancer types had an ORR of 46%. The duration of response was over 6 months in 78% of patients, including 7% complete response (CR) [6]. The updated analysis of KN-158 evaluated 233 patients across 27 cancer types (excluding CRC). Patients were followed for a median period of 13.4 months and demonstrated an ORR of 34.3%, a median progression free survival (mPFS) of 4.1 months, and a median overall survival (mOS) of 23.5 months. The discontinuation rate due to treatment-related adverse effects (TRAEs) was 9.4% and one treatment-related death occurred due to pneumonia [7]. The characteristics of the cancer (location and tumor type), pre-treatment, and other factors may contribute to the efficacy. However, more research needs to be completed to determine which specific factors contribute.

The most common cancer types in the KN-158 efficacy population include malignancies of the reproductive and gastrointestinal systems. Patients with primary brain cancers did not respond to pembrolizumab [7]. Consequently, the FDA included a “limitation of use” for pediatric patients with *MSI-H* brain tumors (FDA, 2017 [5]). Temozolomide treatment in glioblastoma patients can induce therapeutic pressure and MSH-6 mutations, leading to intratumor heterogeneity and potentially reducing the immunotherapy efficacy. Recurrence may also result from treatment-induced hypermutation [13,14]. Although some patients with high-grade glioma improved with treatment, not all have benefited despite the high mutation burden [15,16,17]. Testing for de novo MSI-H/dMMR markers in brain tumor samples could identify treatment-responsive patients; however, their rarity in gliomas raises concerns about cost-effectiveness.

KEYNOTE-177 is a phase III clinical trial that demonstrates the efficacy of 307 patients with stage IV *MSI-H*/*dMMR* CRC. Pembrolizumab monotherapy (ORR = 43.8%, mPFS = 16.5 months) had superior efficacy and survival compared to chemotherapy (ORR = 33.1%, mPFS = 8.2 months) in the first line setting [2]. In contrast, other KN trials feature a heavily pretreated population. Pembrolizumab also improved the toxicity profile for this cohort (hazard ratio = 0.60, 95% CI 0.45–0.80, *p* = 0.0002). High-grade TRAEs (grade 3 and above) occurred in 22% and 66% of patients in the pembrolizumab and chemotherapy group, respectively. The most common high-grade adverse events were decreased neutrophil count, neutropenia, and diarrhea which resulted in a 14% treatment discontinuation rate [2]. The most common TRAEs include systemic symptoms (e.g., fatigue, fever) and gastrointestinal side effects (e.g., diarrhea, nausea, abdominal pain, decreased appetite, vomiting, constipation). There were also reports of immune-mediated adverse events (1–3%) such as colitis, hepatitis, and infusion reactions.

Pembrolizumab was converted from accelerated to full approval on 28 March 2023. This was based on KN-164, -158, and -051. After a median follow-up period of 20.1 months, 504 adult and pediatric patients with over 30 different *dMMR*/*MSI-H* cancers were evaluated. The ORR was 33.3% (95% CI 29.2–37.6), including 10.3% CR and a 23% PR. The response rate among 124 patients with metastatic CRC was 34% (95% CI 26–43) with DOR ranging between 4.4 and 58.5 months. There were also 380 patients with non-CRC that had an ORR of 33% as well as a duration of response ranging between 1.9 and 63.9 months. Overall, patients had a median duration of response of 63.2 months. Approximately 77% of patients had a DOR lasting 12 months or longer and 39% of patients had a DOR lasting 36 months or longer. This marks pembrolizumab as the first tumor-agnostic treatment to receive full FDA approval.

### 2.2. Pembrolizumab Approved for Patients with Unresectable or Metastatic TMB-H Cancers (16 June 2020)

The tumor mutational burden (*TMB*) is a genomic biomarker that is defined as the number of mutations per megabase of DNA (mut/Mb). These somatic mutations may include point mutations, insertions and deletions (indels), copy number alterations (CNAs), as well as chromosomal rearrangements. The point mutations encompass synonymous (silent) and non-synonymous (missense) substitutions, the latter resulting in altered amino acid sequences and neoantigen generation. *TMB-H* tumors with elevated neoantigen levels often have superior responses to immunotherapy [18,19]. No consensus exists on the optimal TMB-H cutoff value, leading to heterogeneous findings in the literature. Methodological variations, such as whole exome sequencing (WES) versus targeted gene panels, and decisions regarding the inclusion/exclusion of synonymous mutations, further contribute to the differences in TMB assessment. One study reviewed the TMB status of 3534 primary tumors from The Cancer Genome Atlas (TCGA) and 696 advanced tumors from Weill Cornell determined by WES [19]. Fernandez and his colleagues determined that a cancer-specific threshold was superior to a pan-cancer threshold approach. In contrast, Chalmers and colleagues used a static TMB threshold of 20 mut/Mb for all cancers. Different cancers exhibit varying TMB values, likely due to differences in mutational rates. For example, prostate cancer’s TMB ranges from 0.30 to 14.13 mut/Mb in TCGA, while bladder cancer ranges from 0.4 to 99.68 mut/Mb [18]. Reducing the cutoff would be overly stringent, while raising it might be too lenient. Therefore, employing a cancer-specific cutoff appears to be a promising approach, especially for rare cancers. In addition to cancer type, the TMB may vary due to patient demographics, treatment intensity, and exogenous factors like smoking and UV radiation.

Pembrolizumab was approved for advanced *TMB-H* positive cancers on 16 June 2020, based on the phase II KEYNOTE-158 trial (Table 1). Tissue *TMB-High* (tTMB-H) was defined as cancers with at least 10 mut/Mb. The responses were seen across eight cancer types (Figure 2). ORR was achieved in 29.4% and 6.3% in the tTMB-H group (*n* = 102) and the non-tTMB-H group (*n* = 688), respectively [8]. Despite a median TMB below the 10 muts/Mb cutoff, responders had a higher median TMB than non-responders. This implies that TMB alone may not fully predict treatment response, as individuals below the cutoff still responded, albeit with higher *TMB* correlating with better response rates.

Pembrolizumab has limited use for *TMB-H* positive central nervous system (CNS) cancers since the efficacy and safety data for this subset of patients have not been established (FDA). Patients with anal cancer also had a poor response rate. Among the 14 patients with *TMB-H* anal cancer, only 1 responded (ORR = 7%), whereas 9 out of 84 patients in the non-*TMB-H* cohort responded [10].

These findings may be attributed to several factors. For example, three patients lacked TMB information, while five patients exhibited TMB ≥ 13 mut/Mb. Given the small sample size, it is challenging to ascertain if these discrepancies stem from the specific TMB distribution in this cancer type or from data constraints. In the safety analysis of 105 patients, common TRAEs included fatigue, asthenia, hypothyroidism, and reduced appetite. Approximately 10% of patients encountered severe TRAEs. Eight patients discontinued treatment, while thirty-four patients had treatment interruptions. Fatal pneumonia related to treatment occurred in one patient with progressive disease [8].

Merck and colleagues also presented a retrospective analysis at the 2020 AACR meeting; however, the results have not been published. A pooled cohort of 2234 patients from 12 studies (KEYNOTE-001, 002, -010, -012, -028, -045, -055, -059, -061, -086, -100, 199) were analyzed using WES. TMB status was determined in 1772 patients who received pembrolizumab monotherapy and 452 patients who received chemotherapy. Among those who received pembrolizumab monotherapy, F1CDx analysis found that 433 patients (24%) had TMB ≥ 175 mut/exome which was roughly analogous to TMB ≥ 10 mut/Mb [10]. The FDA also reviewed three randomized control trials for progression-free survival and overall survival among pembrolizumab monotherapy and chemotherapy cohorts as part of an exploratory post hoc analysis. The cancer types studied were non-small cell lung cancer (NSCLC), urothelial cancer, and gastric cancer from the KEYNOTE-010, KEYNOTE-045, and KEYNOTE-061 trials, respectively.

The FDA has acknowledged the inconsistencies and limitations of TMB; however, the accelerated approval of pembrolizumab for *TMB-H* solid tumors has been beneficial in patients who otherwise cannot receive immunotherapy. Although a TMB cutoff of ≥10 mut/Mb was used for FDA approval, Merck and colleagues also analyzed 70 patients with TMB ≥ 13 mut/Mb that demonstrated an ORR of 37% (95% CI 26–50) and 32 patients with TMB < 13 mut/Mb but ≥10 mut/Mb that demonstrated an ORR of 13% (95% CI 4–29) [10]. A postmarketing study is underway in order to better understand the efficacy and clinical significance behind these different cutoffs.

### 2.3. Dostarlimab Approved for Patients with Unresectable or Metastatic dMMR Cancers (17 August 2021)

Dostarlimab is an IgG4-k monoclonal antibody with activity against PD-1. Dostarlimab monotherapy received accelerated FDA approval for recurrent and/or advanced *dMMR* cancers on 17 August 2021 (Table 1) [20]. This approval was based on the phase I GARNET trial, consisting of several expansion cohorts. These included patients with *dMMR*/*MSI-H* endometrial cancer (EC, cohort A1), *MMR*-proficient (*MMRp*)/microsatellite stable (*MSS*) EC, NSCLC, and non-EC *dMMR*/*MSI-H* and cancers with the *POLE* mutation. The study also included patients with advanced, relapsed, high-grade serous, endometrioid, or clear cell ovarian, fallopian tube, or primary peritoneal cancer (PROC) without known *BRCA* mutation. The FDA approval was based on 209 patients across 16 different cancer histologies (Figure 2). The overall ORR was 41.6% (95% CI 34.9–48.6), including a 9.1% complete response rate and a 32.5% partial response rate. The mDOR was 34.7 months with the vast majority (>95%) of patients experiencing a duration of response for at least 6 months (FDA, 2021 [11]). In an updated analysis, 327 patients were evaluated. The most common cancer types were EC (*n* = 143, ORR = 45.5%) and colorectal cancer (*n* = 115, ORR = 43.5%). Patients also demonstrated a promising mPFS of 6.9 months (Andre et al., 2023). The most common all-grade adverse reactions were fatigue, anemia, and gastrointestinal side effects. The most common high-grade adverse reactions were anemia, fatigue, increased LFTs, sepsis, and acute kidney injury. Dostarlimab toxicity resulted in 25 treatment discontinuations and two deaths [12].

Dostarlimab has heterogenous response rates dependent on cancer histology. In the EC subgroup, the ORR is often >40%. Oaknin and colleagues demonstrated that patients with *dMMR*/*MSI-H* EC (cohort A1, *n* = 143) had superior efficacy compared to patients with proficient (*MMRp*)/*MSS* EC (cohort A2, *n* = 153). Cohort A1 and cohort A1 had a response rate of 45.5% (95% CI 37.1–54.0) and 15.4% (95% CI 10.1–22.0), respectively [12,20]. In contrast, 67 patients with *dMMR* NSCLC had an ORR of 27% with an mDOR of 11.6 months. Patients were further characterized by PD-L1 expression. This was measured by the tumor proportion score (TPS), as follows: <1% (negative), 1–49% (low), ≥50% (high), or unknown status. The TPS status correlated with a positive trend in ORR. Those with a high TPS status (*n* = 5) had immune-related ORR 40%, 0% CR, 12.5% PR, and 80% DCR. Of note, those with unknown status (*n* = 18) had a higher ORR (44.4%) and DCR (72.2%) compared to other subgroups. There were no complete responders; however, eight patients had a partial response [21]. The group with negative status had the greatest proportion of patients, which reflects the fact that a large proportion of patients with NSCLC do not have PD-L1 status. Patients with non-squamous histology also experienced superior efficacy compared to patients with squamous histology. Therefore, biomarker status and cancer subtype contribute to dostarlimab efficacy. The most commonly reported any-grade TRAEs were fatigue, hypothyroidism, and gastrointestinal symptoms (nausea, diarrhea, decreased appetite). There were eight patients (11.9%) who experienced high-grade TRAEs with fatigue being the most common. Overall, treatment was interrupted in 16 patients (23.9%) and discontinued in 4 patients (6%). The most common reasons for discontinuation were due to increased lipase (1.5%), pleural effusion (1.5%), pneumonitis (1.5%), and increased transaminases (1.5%) [21].

In patients with *dMMR*/*MSI-H* positive or *POLE* hypermutated non-EC (cohort F), gastrointestinal cancer compromised 93.4% of this cohort (56% colorectal cancer, 11% gastric cancer). Among 204 patients, 43.1% responded (95% CI 36.2–50.2) with a median follow-up time of 27.7 months. The median PFS was 7.1 months, although the mDOR and mOS were not reached [22]. In regards to toxicity, 68.8% experienced TRAEs. Although these are only interim analyses for the different cancer subtypes, the different expansion cohorts have demonstrated the tumor-agnostic benefit of dostarlimab. Furthermore, patients with *dMMR*/*MSI-H* rectal cancer are usually poor responders to standard therapies such as chemotherapy. A recent phase II trial enrolled 16 patients with *dMMR*, locally advanced rectal cancer that had a minimum follow-up of 6 months. The majority of these patients had stage III rectal cancer (15 patients), whereas only 1 patient had stage II cancer. Dostarlimab reported 100% remission (95% CI 74–100) for 12 patients in June, 2022. The trial shows that 81% of patients had a resolution of their symptoms within 9 weeks of starting treatment [23]. Although 75% of patients experienced any-grade adverse events (the most common being rash or dermatitis, pruritus, fatigue, and nausea), no grade 3/4 adverse events were reported. This shows promising results, especially when compared to the relatively poor response of CRC to other PD-L1 inhibitors. However, it is too early to determine if the efficacy of dostarlimab in this subset of patients can be replicated or if this is due to chance alone.

## 3. Approved Targeted Cancer Therapeutics in the Subject of Histology-Agnostic Drug Development

### 3.1. Larotrectinib Approved for Patients with Unresectable or Metastatic NTRK Gene Fusion-Positive Cancers (26 November 2018)

The neurotrophic tropomyosin receptor kinase (*NTRK*) family consists of three main genes: *NTRK1*, *NTRK2*, and *NTRK3*. These genes code for the TRKA, TRKB, and TRKC transmembrane proteins, respectively. When neurotrophins bind to TRK receptors, dimerization of the receptor consequently occurs. This starts a series of phosphorylation events which activates the *MAPK* pathway (from activated *TRKA*) and the *PI3K*/*AKT* pathway (from activated *TRKC*) [24]. Genetic alterations result in defective receptors leading to constitutively active kinase activity. These fusions primarily stem from chromosomal rearrangements such as deletions, inversions, and translocations. *NTRK* fusions, detected in less than 1% of solid tumors, have a high frequency in rare malignancies (>90%) and a low frequency in common malignancies (>5%) [25]. These rare malignancies include infantile fibrosarcoma, secretory carcinomas of the breast, and salivary gland cancers. This significantly impacts patient enrollment in clinical trials and the ability to gather a sufficient number of patients for evaluating efficacy.

**Figure 3:**
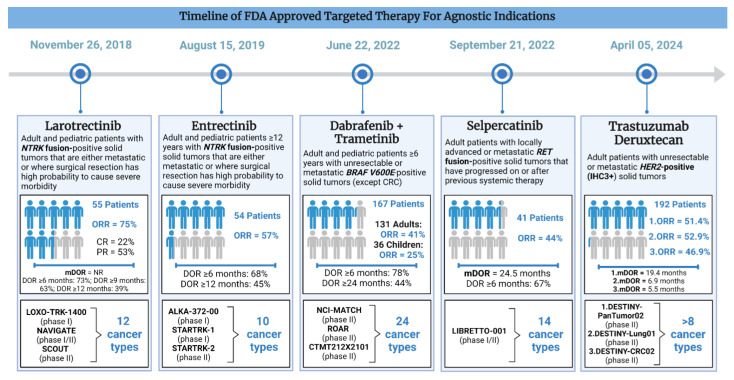
Timeline of FDA-approved targeted therapy for tissue-agnostic indications.

Larotrectinib [LOXO-101] is a selective pan-NTRK inhibitor that was FDA approved for advanced *NTRK* fusion-positive cancers on 26 November 2018 (Table 2). Its approval was on the basis of three phase I/II trials: LOXO-TRK-14001 (adults), SCOUT (children), and NAVIGATE (adults and children). Although 176 patients were enrolled, only the first 55 patients were evaluated. There were 17 unique cancer types including salivary gland cancer (22%), non-gastrointestinal stromal tumor soft tissue sarcoma (non-GIST STS) (20%), infantile fibrosarcoma (13%), thyroid cancer (9%), CRC (7%), NSCLC (7%), and melanoma (7%) (Figure 3) (FDA, 2018 [26]). Larotrectinib was given at an oral dose of 100 mg twice daily in adults. In the pediatric population, the dose was weight-based. A central assessment demonstrated ORR of 75% (95% CI 61–85), including 13% CR and 62% PR [27]. The mDOR was 35.2 months and the mPFS was 25.8 months [28]. The vast majority of patients had a response duration of 6 months or longer (73%), although 39% of patients had a response for at least one year. Based on the safety data from 176 patients (including 44 pediatric patients), the most common all-grade TRAEs include elevated liver enzymes (45%), anemia (42%), neutropenia (23%) fatigue (37%), nausea (29%), vomiting (26%), constipation (23%), diarrhea (22%), and cough (26%). Over half of the cohort also experienced neurological TRAEs, including dizziness (28%). The most common high-grade TRAEs were anemia (10%), neutropenia (7%), elevated liver enzymes (6%), weight gain (4%), and fatigue (3%). CNS and liver toxicities were also included as labeled warnings and 15% of 55 patients had dose reductions due to elevated LFTs, dizziness, and neutropenia. Notably, no treatment-related discontinuations or deaths occurred [28].

Since the FDA approval of larotrectinib, new data have emerged among the ongoing trials. Hong and colleagues looked at non-primary CNS *NTRK* fusion-positive cancers in 194 adults across 24 unique cancer types. A total of 180 evaluable patients had a response rate of 57% (95% CI 50–65), including 26 complete responders (16%) and 74 partial responders (41%). The cancer types included lung cancers (15%), soft tissue sarcomas (155), thyroid cancers (14%), salivary gland cancers (13%), and CRC (12%). The vast majority of patients harbored *NTRK1* and *NTRK3* gene fusions, while <5% had *NTRK2* alterations. Patients were followed for an average of 32.3 months with an mPFS of 24.6 months. Of those who responded, 39 patients had stable disease (22%) while 23 patients progressed (13%). The toxicity data were in line with previously established data, although there was one case of treatment interruption attributed to elevated LFTs [35].

Only one patient out of fifty-five (2%) had brain metastasis at baseline with no intracranial response recorded [36]. In this updated analysis, there were also 22 evaluable patients with baseline CNS metastasis where 68% responded to treatment (95% CI 45–86) [35]. Despite the lower incidence rates of *NTRK* gene fusion status in patients with advanced CNS tumors, there are still unmet needs for this subset of the population. This is especially true for pediatric patients since CNS tumors are a large cause of malignancy-related mortality. Mangum and Parsons found that the intracranial ORR in pediatric patients was 38% with three complete responders and seven partial responders [37]. More research is required to evaluate the true benefit of larotrectinib in these patients.

### 3.2. Entrectinib Approved for Patients with Unresectable or Metastatic NTRK Gene Fusion-Positive Cancers (15 August 2019)

Entrectinib is a multikinase inhibitor which targets *NTRK*, c-ros oncogene 1 (*ROS1*), and anaplastic lymphoma kinase (*ALK*) proto-oncogene tyrosine protein kinase. Entrectinib was FDA-approved for advanced or metastatic *NTRK* fusion-positive cancers in adult and pediatric patients 12 years or older on 15 August 2019 (Table 2). It was based on the results of three clinical trials: ALKA-372-001 (phase I), STARTRK-1 (phase I), and STARTRK-2 (phase II). Patients received a daily oral dose of 600 mg until disease progression or unacceptable toxicity. The efficacy study had a total pool of 54 adult patients across 10 unique cancer types and over 19 different histologies (Figure 3). The most common cancer types were sarcomas (*n* = 13), NSCLC (*n* = 10), MASC (*n* = 7), and breast cancer (*n* = 6). Among 54 evaluable patients, 57.4% responded (95% CI 43.2–70.8), including 7% CR and 50% PR after a median follow-up period of 12.9 months. The majority of patients had a durable response with mDOR of 10.4 months (95% CI 7.1-NE), including 68% (among 31 responders) with a DOR ≥ 6 months and 45% with a DOR ≥ 12 months. Entrectinib also demonstrated a survival benefit with mPFS of 11.2 months (95% CI 8–14.9) and mOS of 21 months (14.9-NE) [38]. The most common all-grade TRAEs include gastrointestinal and neurological side effects as well as edema, shortness of breath, and myalgia. The most common high-grade TRAEs among the 68 patients include weight gain (10%), anemia (12%), as well as CNS TRAEs such as dizziness (4%) and cognitive changes (3%). TRAEs resulted in treatment interruptions (46%), dose reductions (29%), and discontinuations (9%). However, no treatment-related deaths occurred [30].

An updated analysis evaluated 121 adult patients across 14 unique cancer types and over 30 different histologies for a median follow-up of 25.8 months. The overall ORR was 61.2% (95% CI 51.9–69.9), including 19 complete responders and 55 partial responders. There were also improvements in the mDOR (20 months, 95% CI 13–38.2), mPFS of (13.8 months, 95% CI 10.1–19.9), and mOS (33.8 months, 95% CI 23.4–46.4) [39]. While cohorts had response rates > 50%, CRC patients (*n* = 10) had an ORR of only 20%. However, the mDOR was 17.6 months, suggesting that a small subset of patients still benefit.

On 20 October 2023, the approval of entrectinib was expanded to include pediatric patients as young as 1 month. This was on the basis of the phase I/II STARTRK-NG trial and the phase II TAPISTRY trial. The most common cancer types studied were primary CNS tumors and infantile fibrosarcoma. Among 33 pediatric patients with *NTRK* fusion-positive cancers, the ORR was 70% (95% CI 51–84) with an mDOR of 25.4 months (95% CI 14.3-NE) (FDA, 2023 [29]). A safety analysis of 76 pediatric patients indicated tolerability of entrectinib, with gastrointestinal adverse events being the most common.

Unlike larotrectinib, entrectinib was designed to have more CNS penetrating efficacy. The updated integrated analysis demonstrated an intracranial ORR of 63.6% (95% CI 30.8–89.1) among 11 patients who had baseline brain metastasis [39]. This is potentially due to its ability to remain in the CNS as a weak p-glycoprotein (P-gp) substrate compared to larotrectinib and crizotinib which have stronger interactions with P-gp substrates [40]. P-gp has an important role at the blood–brain barrier and entrectinib’s weak interaction with the efflux transporter allows for greater CNS distribution. Entrectinib can therefore meet some of the unmet needs for patients with *NTRK* gene fusion-positive cancers that have metastasized to the brain.

Several challenges include the heterogeneity of fusion partners, the plethora of diagnostic tools, and differing guidelines. Serious TRAEs may also not be clinically observable due to these low numbers, so more safety analyses are required to better understand these toxicities. Both larotrectinib and entrectinib are still undergoing postmarketing analysis, with the potential use of in vitro companion diagnostic devices to select patients for treatment. Although there are currently two FDA approvals targeting *NTRK* fusion-positive cancers, the detection and treatment of these patients can still be challenging due to the rarity of these alterations.

### 3.3. Dabrafenib plus Trametinib Approved for Patients with Unresectable or Metastatic BRAF^V600E^ Positive Cancers (22 June 2022)

B-raf proto-oncogene serine/threonine kinase (BRAF) is a key enzyme in the mitogen-activated protein kinase (MAPK) signaling pathway. BRAF phosphorylates and activates downstream enzymes such as MEK and ERK. *BRAF* mutations (the vast majority comprising *BRAF^V600E^*) cause the hyperproliferation of the pathway, resulting in tumor cell growth. *BRAF* mutations are present in melanoma (50%), NSCLC (1–5%), anaplastic thyroid carcinoma (20–50%), biliary tract cancer (5–7%), low-grade gliomas (5–15%), and glioblastomas (3%) [41]. They have also been found in select cases of hairy cell leukemia, Langerhans cell histiocytosis, and CRC. The previous trials have demonstrated responses to BRAF monotherapy (such as vemurafenib); however, resistance quickly emerged. One of the known resistance mechanisms is via downstream activation (“on-target” resistance), leading to the hyperactivation of the MAPK signaling pathway regardless of BRAF inhibition. To mitigate this, MEK inhibitors were added.

Dabrafenib (BRAF inhibitor) and trametinib (MEK inhibitor) were approved for unresectable or metastatic *BRAF^V600E^* positive solid tumors in adults and pediatric patients ≥ 6 years on 22 June 2022 (Table 2). However, colorectal cancers were an exception to this indication due to known resistance. The approval was based on three trials: the National Cancer Institute-Molecular Analysis for Therapy Choice (NCI-MATCH) and Rare Oncology Agnostic Research (ROAR) trials which evaluated adult patients, as well as CTMT212X2101 (X2101) which evaluated pediatric patients. This was supported by results from COMBI-d, COMBI-v, and BRF113928 (FDA, 2022, [31]). The NCI-MATCH trial was the first national study in the United States to integrate a unified method of diagnosis despite the diverse geographical locations. A total of 5954 patients with refractory malignancies were enrolled across a wide network of 1117 participating locations [42]. The second trial, ROAR, was a phase II study with a specific focus on patients with rare cancers carrying the *BRAF^V600E^* mutation. Lastly, the CTMT212X2101 study (part C and part D) focused on pediatric patients with gliomas. The data for FDA approval comprised a total of 167 evaluable patients (131 adults and 36 children) across 24 unique cancer types (Figure 3). The ORR was 41% (95% CI 33–50) and 25% (95% CI 12–42) among adult and pediatric patients, respectively. Responses were durable, with 78% having a DOR 6 months or longer and 44% having a DOR 24 months or longer. The most common cancers in adults were biliary tract cancer (*n* = 48, ORR = 46%), high-grade glioma (*n* = 48, ORR = 33%), low-grade gliomas (*n* = 14, ORR = 50%), and various malignancies of the gastrointestinal tractThe most common TRAEs were nausea, constipation, vomiting, pyrexia, fatigue, rash, peripheral edema, headache, cough, and hemorrhage.

Corcoran and colleagues demonstrated that 43 patients with metastatic CRC had an ORR of only 12%. Responders often harbored *PIK3CA* co-mutations. Although the mPFS was 3.5 months, this still surpasses the survival outcomes from standard chemotherapy [43]. CRC has historically been difficult to target, with many hypothesizing that this is due to “off-target” resistance from *EGFR* activation. Thus, encorafenib (BRAF inhibitor) plus cetuximab (EGFR inhibitor) are currently approved for these *BRAF^V600E^*-positive metastatic CRCs (FDA, 2020, [44]). There are also clinical trials assessing triple therapy. For example, dabrafenib plus trametinib plus spartalizumab exhibited a 24.3% response rate [41]. However, the potential risk of increased toxicity must be evaluated in these patients.

Dabrafenib plus trametinib also received FDA approval for children with *BRAF^V600E^*-positive low-grade gliomas on 16 March 2023. This was based on the low-grade glioma sub-cohort from the TADPOLE trial (FDA, 2023 [45]) [46]. Among 110 patients (73 receiving dabrafenib and trametinib therapy), the ORR was 46.6%, the mDOR was 23.7 months, and the mPFS was 20.1 months. Unfortunately, there is still no approval for *BRAF^V600E^*-positive high-grade gliomas for patients less than 6 years of age. Notably, the pooled data from the NCI-MATCH and ROAR trials demonstrated that patients with high-grade gliomas (*n* = 48) had the lowest response rate (ORR = 33%) as well as an mDOR of 3.9 months. However, a post hoc analysis found that stratifying patients by age (18–39 years) improved the objective response rate to 50%, similar to the low-grade glioma cohort [41]. Although only 5–10% of pediatric high-grade gliomas harbor a *BRAF^V600E^* mutation, there is a high mortality associated with these malignancies. This represents the clinical benefit of these medications among young adults compared to the pediatric population.

### 3.4. Selpercatinib Approved for Patients with Unresectable or Metastatic RET-Positive Cancers in Patients ≥ 12 Years (21 September 2022)

The rearranged during transfection (*RET*) gene encodes proto-oncogenic transmembrane receptor tyrosine kinases (RTKs). They are activated by binding to the glial cell-line-derived neurotrophic factor (GDNF) ligands. This forms a heterodimeric ligand–co-receptor complex, which initiates signaling cascades crucial for cellular growth and differentiation. This pathogenesis is believed to arise from chromosomal instability, leading to gene fusions with partners such as *KIF5B*. *RET* gain-of-function mutations are implicated in malignancies such as invasive breast cancers (30–70%), pancreatic ductal adenocarcinomas (50–60%), sporadic papillary thyroid carcinomas (2.5–73%), and NSCLC (1–3%) [47]. They have also been reported in Hirschsprung’s disease (loss-of-function mutations) and MEN2 syndrome (inherited form). *RET* fusions have a frequency of <1% across multiple cancers. They are prevalent in rare cancers such as MASC, while they are rare in common cancers.

Selpercatinib [LOXO-292/LY3527723], a potent RET inhibitor with CNS-penetrating activity, gained orphan drug status due to the lack of effective targeted therapies [48]. On 21 September 2022, selpercatinib was granted FDA approval for *RET* fusion-positive advanced solid tumors in adult and pediatric patients (Table 2). This was based on the ongoing phase I/II trial LIBRETTO-001, spanning 30 sites across eight countries [49]. Patients weighing <50 kg received 120 mg and those ≥ 50 kg received 160 mg tablets, taken twice daily for a maximum of 2 years. The efficacy population consisted of 41 patients across 14 unique cancer types, excluding NSCLC and thyroid cancer (Figure 3). Pancreatic adenocarcinoma (27%), CRC (24%), salivary cancer (10%), and cancers of unknown primary (7%) were the most common. The response rate was 43.9% (95% CI 28.5–60.3), including two complete responders and sixteen partial responders. Patients had an mPFS of 13.2 months, an mOS of 18 months, as well as an mDOR of 24.5 months. The majority of patients had a response for at least 6 months (>65%) (FDA, 2022 [32]). The safety population included 796 patients. The most commonly reported toxicities were diarrhea, constipation, nausea, fatigue, dry mouth, hypertension, rash, and headache. The most common serious TRAEs were elevated hypertension in 22% of patients, as well as elevated ALT and AST in 16% and 13% of patients, respectively [49].

After 16 months of further follow-up, selpercatinib continued to have durable responses in *RET* fusion-positive gastrointestinal cancers. Patients with pancreatic cancer (*n* = 13) had a response rate of 53.8% (95% CI 25.1–80.8), including one complete responder and six partial responders. Responses were durable with an mDOR > 50 months and mPFS was 5.6 months. Patients with colorectal cancer (*n* = 13) had an ORR of 30.8% (95% CI 9.1–61.4), an mDOR of 13.3 months, as well as an mPFS of 9.1 months [50]. CRC and pancreatic cancer face efficacy challenges due to resistance. *RET* fusions are also rare in both cancer types, posing diagnostic challenges. A significant number of CRC patients also had *MSI-H* status, suggesting that some may benefit from combinational therapies. This underlines the importance of genomic testing and their role in treatment.

The FDA approval was supported by 343 patients with *RET* gene fusion-positive NSCLC and thyroid cancer in the same trial. Gautschi and colleagues reported the final data from LIBRETTO-001 for patients with *RET* fusion-positive NSCLC. Among 249 pretreated patients, the ORR was 62% (95% CI 55–68) with an mDOR of 31.6 months (95% CI 20.4–42.3). Patients also had a survival benefit with mPFS of 26.2 months (95% CI 19.3–35.7) and mOS of 47.6 months (95% CI 35.9-NE) [51]. Similarly, 19 patients with *RET* fusion-positive thyroid cancers demonstrated an impressive response rate of 79% (95% CI 54–94) [52]. Selpercatinib was approved for *RET*-mutant medullary thyroid cancers; however, no approvals currently exist for *RET*-mutant solid tumors. Notably, the ORR for other cancer types is significantly lower. However, this may be due to the prevalence of gastrointestinal cancers included in the efficacy population.

### 3.5. Trastuzumab Deruxtecan Approved for Patients with Unresectable or Metastatic HER2-Positive Cancers (5 April 2024)

Human epidermal growth factor receptor 2 (HER2) is encoded by the *ERBB2* gene and is one of four receptor tyrosine kinases in the HER family (HER1-4). Activated *HER2* can interact with MAPK and PI3KT/AKT pathways, promoting tumor cell growth. HER2 positivity has been reported in bladder cancer (16%), esophageal cancer (14.9%), breast cancer (18.3%), gallbladder cancer (11.11%), cholangiocarcinoma (8.5%), gastric adenocarcinoma (17.3%), ovarian cancer of epithelial origin (8.16%), and various head and neck cancers (4–19%) [53]. The *HER2* expression is often measured by the immunohistochemistry (IHC) score. This includes IHC 3+ (HER2-positive), IHC2+ (borderline), and IHC 0–1+ (HER2-negative).

Fam-trastuzumab deruxtecan-nxki or trastuzumab deruxtecan (T-DXd) is an anti-HER2 antibody drug conjugate (ADC) which consists of an HER2 antibody, a linker, and a cytotoxic agent (topoisomerase I inhibitor) (Dxd) [54]. A meta-analysis demonstrated significant efficacy and survival benefits compared to standard therapy [55]. On 5 April 2024, T-Dxd was granted accelerated approval for unresectable or metastatic HER2-positive (IHC 3+) solid tumors which had received previous systemic treatment and for which there were no suitable alternative options (Table 2). A total of 192 patients were involved across three trials: DESTINY-PanTumor02 (ORR = 51.4%, mDOR = 19.4 months), DESTINY-Lung01 (ORR = 52.9%, mDOR = 6.9 months), and DESTINY-CRC02 (ORR = 46.9%, mDOR = 5.5 months) (Figure 3) (FDA, 2024 [33]).

DESTINY-PanTumor02 is a phase II multicohort trial featuring a heterogeneous cohort with IHC2+/3+ histologies across 15 countries. A total of 267 patients were evaluated over a median follow-up period of 9.7 months. The objective response rate was 37.1% (95% CI 31.3–43.2) with an mDOR of 11.8 months. Patients with reproductive solid tumors (endometrial, cervical, and ovarian) had the highest response rates (≥45%), whereas other cancer types had an ORR < 40% [34,56]. Notably, 25 patients with pancreatic cancer had an ORR of 4.0%. The first 15 patients with pancreatic cancer did not respond and recruitment was consequently discontinued. Similarly, patients with HER2-positive metastatic CRC (DESTINY-CRC02) had poor ORR (<40%), including a 0% complete response rate [57]. Patients with HER2-overexpressed metastatic NSCLC (DESTINY-Lung01) received either 6.4 mg/kg (cohort 1) or 5.4 mg/kg (cohort 1A). Patients in cohort 1 (*n* = 49) had a response rate of 26.5% (95% CI 15.0–41.1) and an mDOR of 5.8 months (95% CI 4.3-NE). Similarly, patients in cohort 1A had a response rate of 34.1% (95% CI 20.1–50.6) and an mDOR of 6.2 months (95% CI 4.2–9.8). Both groups demonstrated an impressive disease control rate (>65%) [58]. In contrast, patients with *HER2*-mutant metastatic NSCLC (cohort 2) were evaluated in the DESTINY-Lung02 trial. Those who received T-Dxd 5.4 mg/kg and 6.4 mg/kg had an ORR of 49.0% (95% CI 39.0–59.1) and 56.0% (95% CI 41.3–70.0), respectively. Both groups also had remarkable disease control rates (>90%) [59]. These findings suggest that characterizing via molecular subtype (HER *2*-overexpression vs. *HER2*-mutation) is clinically relevant.

The FDA reported the most common (≥20%) TRAEs involved cytopenia of three cell lines (leukocytes, erythrocytes, and platelets), gastrointestinal side effects, as well as decreased electrolytes (FDA, 2024 [33]). In DESTINY-PanTumor02, 58.7% experienced grade 3 TRAEs. This also contributed to treatment discontinuation in 11.6% of patients [34,56]. Three deaths were attributed to interstitial lung disease. Notably, patients with a past medical history of interstitial lung disease and/or pneumonitis (either requiring steroid treatment or a diagnosis of significant cardiac disease at screening) were excluded. Similarly, patients with lung cancers had an increased risk of treatment-related ILD. The NSCLC cohort had a significant ILD rate of 26%. Patients who received the FDA-recommended dose (5.4 mg/kg) and those who had a higher dose (6.4 mg/kg) had a treatment-related ILD rate of 20% and 5%, respectively [58]. This warrants careful monitoring and screening for pulmonary injury. Early detection and management is vital to preventing fatal respiratory toxicity.

In DESTINY-PanTumor02, patients with IHC 3+ (HER2 positive) cancers had a more favorable objective response compared to IHC 2+ (borderline) cancers. The pan-cancer ORR was 61.3% versus 27.2%, respectively [34,56]. The options for cancers with lower *HER2* expression have limited treatment options. However, a subset of patients with HER2 negative cancers may still benefit from T-DXd. Currently, diagnostic guidelines and recommendations have been established in HER2-positive and HER2-low breast cancer. However, this has not been established for other cancer types. T-DXd is the first ADC to be granted agnostic approval for HER2 IHC3+ cancers. T-DXd’s approval for HER2 IHC3+ cancers mark the first ADC to receive agnostic approval, potentially opening avenues for future ADC agnostic approvals. Accurate pan-cancer diagnostic recommendations are essential for identifying the patients most likely to respond.

## 4. Biostatistics—Trial Design and Conducting in Tissue-Agnostic Drug Approvals

Biostatistics plays a role in designing and conducting clinical trials and has had a significant impact on cancer research and new drug developments. Traditional oncology clinical trials are designed to estimate the average effect, often derived from randomized clinical trials of unselected patients with all-comers. This has been the mainstay of drug approvals for decades. Recent tissue-agnostic clinical trials that utilize molecular genetics and biomarker-driven strategies have made a significant paradigm shift in precision cancer medicine (Figure 2 and Figure 3). As a result, biomarker-driven, tissue-agnostic indications have seen approval by the US FDA (Figure 1). This paradigm shift raises some issues regarding biostatistical inference and study design. Berry [60] stated, “it is ironic that we take the same clinical trial approach to evaluate all manner of potentially amazing transformative experimental therapies and yet we don’t experiment with the design of the clinical trial itself”. There have been remarkable advances in the development of molecularly targeted agents. However, the modernization of clinical trial designs has been outpaced. Now, alternative designs have emerged to catch up to these advances. There are two main approaches for trial design. One approach is an all-comers design that treats all patients with the specific disease and determines the indication through subgroup analysis. The other approach is an enrichment design that enrolls limited patients with the therapy’s targeted genomic or molecular aberrations. Some novel enrichment design/adaptive biomarker-driven trials have been developed such as master protocol, basket trial, umbrella trial, and platform trial. These novel designs investigate hypotheses through concurrent multiple treatment arms or populations, allowing for the addition or removal of arms during the trials [61]. A number of authors have noted confusion regarding the definitions of these terms [62,63,64,65], and some authors have offered a taxonomy of trial designs [60,66,67]. Park et al. [61] performed a systematic review of basket trials, umbrella trials, and platform trials [61]. They identified 49 basket, 18 umbrella, and 16 platform trials, and the median sample sizes of the basket and umbrella trials were 205 and 346, respectively. The majority of basket trials were exploratory non-randomized trials, while most umbrella trials were also exploratory but with more use of randomization than basket trials. Notably, basket trials emerged as a strong tool for evaluating biomarker-targeted therapies among multiple tumor histologies. Many of the basket trials were designed to estimate high and durable objective responses and were conventionally conducted within the phase I and phase II setting [61,68]. Only a few phase III studies, ALCHEMIST (NCT02193282, NCT02595944, and NCT02201992) and CLUSTER (NCT02059291), employed a basket design. Despite the emergence of novel trial designs, there are remaining challenges for designing and performing clinical trials. In 2022, the FDA released a guidance document describing “Tissue Agnostic Drug Development in Oncology”. Adequate justification for the number of subjects and cancer types in each trial with a meaningful treatment effect for a tissue-agnostic oncology drug development is required while controlling for Type I error [69]. With basket trials, basket-wise and family-wise type I errors arise [63,70]. The basket-wise type I error rate is for an individual tumor type, while the family-wise type I error rate is a multiplicity adjusted error rate that represents the false positive rate for at least one of the null baskets. Despite the burgeoning number of potential anticancer drugs and increased numbers of people with cancer, fewer clinical investigations are possible because of limited resources [2]. With traditional statistical approaches, it is a challenge to have an adequately powered sample size with strong control of type I error rate for each individual tumor type due to the limited resources. It is becoming common for trials to be tailored to detect enhanced efficacy in a patient subpopulation, and large clinical trials will soon be less possible, if not impossible [71,72]. Several authors such as Menis et al. [63] and Burock et al. [73] have stated that the goal of cancer clinical trials in this era of precision medicine should be to conduct ‘trials designed to learn’ which lead to ‘trials designed to conclude’, which begins with identifying large and meaningful differences within small, molecularly enriched groups of patients, often referred to as ‘home runs’ [74]. With advances in biology, every cancer will become a rare cancer. Statistical approaches will have to evolve to make the best use of limited resources through the use of novel designs. Before the era of these novel designs, the majority of statistical analyses investigated population/marginal-level responses of intervention. In basket trials, the marginal response rate from pooled analysis may increase the statistical power because of the overall sample size. However, heterogeneity and imbalanced enrollment are common among basket trials, and pooled analysis risks erroneous conclusions as outcomes from over-represented tumor types may be extrapolated to individual histology. Basket-independent analysis could address this issue by avoiding the assumption of global effect, but it is limited by sample size and accrual, and one may need to adopt an analysis strategy that controls family-wise type I errors among multiple hypothesis tests [68]. In addition, the importance of bioinformatics, now more than ever, cannot be overstated as molecular profiling of tumor biopsies plays an increasingly important role in cancer research [75]. Appropriate bioinformatic methods for managing and integrating large and complex data are necessary to make possible the statistical analysis needed to discover and validate predictive models based on omics technologies [72].

## 5. Future Directions and Challenges

Examples of agents that are showing promising clinical benefit across tumor types but have not yet received regulatory approval are *FGFR* inhibitors and *KRAS^G12C^* inhibitors.

### 5.1. Targeting Fibroblast Growth Factor Receptor (FGFR)

Fibroblast growth factor receptor (*FGFR*) is a family of transmembrane receptor tyrosine kinases. *FGFR1* (3.5%), *FGFR2* (1.5%), *FGFR3* (2%), and *FGFR4* (0.5%) alterations are present in a wide range of histologies. These include cancers of the breast, lung, and gastrointestinal tract [76]. Erdafitinib is an anti-FGFR 1–3 agent that is being evaluated in the ongoing phase II RAGNAR trial. Among 217 patients with 16 unique cancer types, the ORR was 30% after 17.9 months of follow-up. Erdafitinib showed a survival benefit with mPFS of 4.2 months and mOS of 10.7 months. Approximately 70% of patients experienced grade 3 adverse events, with >8% of these being serious TRAEs. However, no treatment-related deaths occurred [77,78]. Only patients aged 12 or older were enrolled; therefore, efficacy in younger patients is unestablished. *FGFR* alterations had an incidence of 3% of solid tumors across 19 different histologies including rhabdomyosarcomas and gliomas [79]. These cancer types are more prevalent in pediatric patients. The role of pan-FGFR inhibitors in this cohort as well as the general tumor-agnostic avenue requires a larger patient population.

### 5.2. Tackling Kirsten Rat Sarcoma Virus (KRAS^G12C^)

Kirsten rat sarcoma virus (*KRAS*) is a gene that encodes for the K-Ras protein, a crucial aspect of the MAPK signaling pathway responsible for cell survival and apoptosis. It is frequently mutated in CRC (45%), pancreatic cancers (90%), and NSCLC (35%) [80]. *KRAS* has historically been difficult to target due to its complex biological structure and GDP-GTP bound state. The emergence of *KRAS^G12C^* inhibitors, sotorasib and adagrasib, has garnered interest since their approval for NSCLC. Despite their success, there are still many patients with *KRAS*-positive solid tumors that do not have satisfactory therapies. Divarasib (GDC-6036) is a more selective and potent *KRAS^G12C^* inhibitor that has demonstrated improved efficacy. In a recent analysis of a phase 1 trial, 137 patients receiving divarasib were evaluated. The results were promising, revealing a 53.4% confirmed response rate in 60 patients with NSCLC, and a median progression-free survival of 13.1 months. In 55 patients with colorectal cancer, a 29.1% confirmed response rate and a median progression-free survival of 5.6 months were observed. The most common TRAEs were gastrointestinal toxicity, including nausea (74%), diarrhea (61%), and vomiting (58%). Of those who experienced adverse events, four patients discontinued treatment and nineteen required a dose reduction [81]. Only around 13% of solid tumors harbor a *KRAS^G12C^* mutation [80]. There are still unmet needs for patients who harbor alterations of other *KRAS* isoforms, such as *KRAS^G12D^*. Pan-KRAS inhibitors are also currently in development.

## 6. Conclusions

The ability to develop agents for tumor-agnostic indications holds tremendous promise, especially in rare cancers, where it may not be feasible to conduct adequately powered trials. Increasing the utilization of next generation sequencing is identifying the presence of molecular targets in pediatric and adult cancers, allowing enrollment in basket trials. The challenges around trial enrollment, incomplete understanding of the biology of target in the context of underlying histology, development of resistance, tumor heterogeneity, and management of toxicity, remain to be addressed.

## Figures and Tables

**Table 1:** Summary of FDA-approved immunotherapy.

Drug Name	Pembrolizumab	Pembrolizumab	Dostarlimab-Gxly
Mechanism of action	PD-1 inhibition	PD-1 inhibition	PD-1 inhibition
Indications	Adult and pediatric patients with unresectable or metastatic *dMMR*/*MSI-H* positive solid tumors No satisfactory alternative treatments available or progression despite previous treatment OR Patients with dMMR/MSI-H CRC who have progressed with previous treatment (fluoropyrimidine, oxaliplatin, and irinotecan)	Adult and pediatric patients with unresectable or metastatic *TMB-H* (≥10 mut/Mb) solid tumors No satisfactory alternative treatments available or progression despite previous treatment	Adult patients with unresectable or metastatic *dMMR* solid tumors No satisfactory alternative treatments available or progression despite previous treatment
Date of FDA approval	23 May 2017	16 June 2020	17 August 2021
Clinical trial (s)	KN-016 (phase II) KN-164 (phase II) KN-012 (phase II) KN-028 (phase II) KN-158 (phase II)	KN-158 (phase II)	GARNET (phase I)
Recommended regimen	Adults: IV 200 mg every 3 weeks Children: IV 2 mg/kg (maximum 200 mg) every 3 weeks	Adults: IV 200 mg every 3 weeks OR IV 400 mg every 6 weeks Children: IV 2 mg/kg (maximum 200 mg) every 3 weeks	IV 500 mg every 3 weeks (doses 1–4) IV 1000 mg every 6 weeks (3 weeks after 4; dose 5+)
Number of patients (*n*)	149	102	209
Number of unique cancer types	15	9	16
Most common cancer types	CRC, EC, gastric cancer, CCA	SCLC, CC, EC, anal cancer	EC, CRC, gastric/GEJ cancer, small intestinal cancer
Major efficacy/outcomes	ORR = 39.6% (95% CI 31.7–47.9) CR = 11 (7%) PR = 48 (32%) mDOR = NE (95% CI 1.6–22.7) DOR ≥ 6 months: 78%	ORR = 29% (95% CI 21–39) CR = 4% PR = 25% mDOR = NR DOR ≥ 12 months: 57% DOR ≥ 24 months: 50%	ORR = 41.6% (95% CI 34.9–48.6) CR = 9.1% PR = 32.5% mDOR = 34.7 months (95% CI 2.6–35.8+) DOR ≥ 6 months: 95.4%
Most common TRAEs	Systemic (fatigue, fever, pruritus) Gastrointestinal (constipation, diarrhea, nausea, reduced appetite) Respiratory (cough, dyspnea) Immune-mediated (colitis, endocrinopathies, hepatitis, pneumonitis, nephritis) Musculoskeletal (musculoskeletal pain) Dermatologic (rash)	Systemic (fatigue, fever, pruritus, pain) Gastrointestinal (abdominal pain, constipation, diarrhea, reduced appetite, nausea) Respiratory (cough, dyspnea) Immune-mediated (colitis, endocrinopathies, hepatitis, pneumonitis, nephritis) Musculoskeletal (musculoskeletal pain) Dermatologic (rash)	Most common all-grade TRAEs: Systemic (fatigue, asthenia) Gastrointestinal (diarrhea, nausea) Hematological (anemia) Immune-mediated (colitis, endocrinopathies, hepatitis, pneumonitis, nephritis) Dermatologic Most common high-grade TRAEs: General (fatigue, asthenia, sepsis) Hematologic (anemia) Hepatic (increased liver enzymes) Renal (acute kidney injury)
Reference (s)	FDA, 2017 [5] Marabelle et al., 2020 [8]	FDA, 2020 [9] Marcus et al., 2021 [10]	FDA, 2021 [11] Andre et al., 2023 [12]

Abbreviations: Cholangiocarcinoma (CCA); colorectal cancer (CRC); complete response (CR); deficient mismatch repair (*dMMR*); duration of response (DOR); endometrial cancer (EC); gastroesophageal junction cancer (GEJ); intravenous (IV); keynote trials (KN); median duration of response (mDOR); microsatellite instability-high (*MSI-H*); mutations/megabase (mut/Mb); objective response rate (ORR); partial response (PR); programmed cell death protein-1 (PD-1); small cell lung cancer (SCLC); tumor mutational burden-high (*TMB-H*); treatment-related adverse effects (TRAEs).

**Table 2:** Summary of FDA-approved targeted therapy.

Drug Name (s)	Larotrectinib	Entrectinib	Dabrafenib + Trametinib	Selpercatinib	Trastuzumab Deruxtecan
Mechanism of action	*NTRK* fusion inhibition	*NTRK* fusion inhibition	*BRAF* + *MEK* inhibition	*RET* fusion inhibition	HER2 inhibition
Indication	Adult and pediatric patients with *NTRK* fusion-positive solid tumors that are either metastatic or where surgical resection has high probability to cause severe morbidity No known acquired resistance and no satisfactory alternative treatments available or progression after treatment	Adult and pediatric patients ≥ 12 years with *NTRK* fusion-positive solid tumors that are either metastatic or where surgical resection has high probability to cause severe morbidity No known acquired resistance and no satisfactory alternative treatments available or progression after treatment	Adult and pediatric patients ≥ 6 years with unresectable or metastatic *BRAF^V600E^*-positive solid tumors (except CRC) No satisfactory alternative treatments available or progression despite previous treatment	Adult patients with locally advanced or metastatic *RET* fusion-positive solid tumors that have progressed on or after previous systemic therapy No satisfactory alternative treatments available or progression despite previous treatment	Adult patients with unresectable or metastatic HER2-positive (IHC3+) solid tumors No satisfactory alternative treatments available or progression despite previous treatment
Date of FDA approval	26 November 2018	15 August 2019	22 June 2022	21 September 2022	5 April 2024
Clinical trial (s)	LOXO-TRK-1400 (phase I) NAVIGATE (phase I/II) SCOUT (phase II)	ALKA-372-00 (phase I) STARTRK-1 (phase I) STARTRK-2 (phase II)	NCI-MATCH (phase II) ROAR (phase II) CTMT212X2101 (phase II)	LIBRETTO-001 (phase I/II)	DESTINY-PanTumor02 (phase II) DESTINY-Lung01(phase II) DESTINY-CRC02 (phase II)
Recommended regimen	Adult patients: PO 100 mg twice daily Pediatric patients: PO 100 mg/m^2^ (maximum of 100 mg) twice daily	Route: Oral Dose: 600 mg (children ≥ 12 years: dose based on body surface area) Frequency: Once daily	Adult patients: PO dabrafenib 150 mg (given as two 75 mg capsules) twice daily PLUS PO trametinib 2 mg once daily\ Pediatric patients: Weight-based doses *	PO 120 mg twice daily (<50 kg) OR PO 160 mg twice daily (≥50 kg)	IV 5.4 mg/kg every 3 weeks
Number of patients (*n*)	55	54	167 (131 adults, 36 children)	41	192
Number of unique cancer types	12	10	24 (includes different LGG and HGG subtypes)	14	>8
Most common cancer types	SGT, STS, IFS, TC	Sarcoma, NSCLC, MASC, BC, TC, CRC	BTC, HGG, LGG	Pancreatic adenocarcinoma, CRC, SGT, unknown primary * NSCLC and TC excluded	EC, CC, OC, URO, BTC, NSCLC, CRC
Major efficacy/ outcomes	ORR = 75% (95% CI 61–85) CR = 22% PR = 53% mDOR = NR DOR ≥ 6 months: 73% DOR ≥ 9 months: 63% DOR ≥ 12 months: 39%	ORR = 57% (95% CI 43–71) DOR ≥ 6 months: 68% DOR ≥ 12 months: 45%	ORR (adult patients): 41% (95% CI 33–50) ORR (pediatric patients): 25% (95% CI 12–42) DOR ≥ 6 months: 78% DOR ≥ 24 months: 44%	ORR = 44% (95% CI 28–60) mDOR = 24.5 months (95% CI 9.2—NE) DOR ≥ 6 months: 67%	DESTINY-PanTumor02: ORR = 51.4% (95% CI 41.7–61.0), mDOR = 19.4 (1.3–27.9+) months DESTINY-Lung0: ORR = 52.9% (95% CI 27.8–77.0), mDOR = 6.9 (4.0–11.7+) months DESTINY-CRC02: ORR = 46.9% (95% CI 34.3–59.8), mDOR = 5.5 (1.3+–9.7+) months
Most common TRAEs	Systemic (fatigue) Gastrointestinal (constipation, diarrhea, nausea, vomiting) Hepatic (elevated liver enzymes) Neurological (dizziness) Respiratory (cough)	Systemic (fatigue, edema, fever, increased weight) Gastrointestinal (constipation, diarrhea, nausea, vomiting) Respiratory (cough, dyspnea) Neurological (cognitive impairment, dizziness, dysgeusia, dysesthesia) Musculoskeletal (arthralgia, myalgia) Other (vision disorders) Most serious TRAEs: Cardiac (congestive heart failure, prolonged QT) Hepatic (liver toxicity) Neurological (central nervous system effects) Musculoskeletal (skeletal fractures) Other (high uric acid, vision disorders)	Adult patients: Systemic (fever, fatigue, chills, edema) Gastrointestinal (nausea, vomiting, constipation, diarrhea) Respiratory (cough) Hematologic (hemorrhage) Neurological (headache) Musculoskeletal (myalgia, arthralgia) Dermatologic (rash) Pediatric patients: Systemic (fever, fatigue) Gastrointestinal (vomiting, diarrhea, abdominal pain, nausea, constipation) Respiratory (cough) Dermatologic (dry skin, rash, dermatitis acneiform) Neurologic (headache) Hematologic (hemorrhage) Other (paronychia)	Systemic (edema, fatigue, dry mouth, hypertension) Gastrointestinal (diarrhea, abdominal pain, constipation, nausea) Neurological (headache) Dermatologic (rash)	Systemic (fatigue) Hematological (decreased lymphocytes, platelets, and erythrocytes) Gastrointestinal (vomiting, decreased appetite, diarrhea, constipation, stomatitis) Hepatic (elevated liver enzymes) Respiratory (upper respiratory tract infection) Dermatologic (alopecia) Other (elevated alkaline phosphatase, decreased potassium and sodium)
Reference (s)	FDA, 2018 [26]	FDA, 2019 [29] Doebele et al., 2020 [30]	FDA, 2022 [31]	FDA, 2022 [32]	FDA, 2024 [33] Meric-Bernstam et al., 2023 [34]

Abbreviations: Biliary tract cancer (BTC); bladder cancer (URO); breast cancer (BC); B-Raf proto-oncogene serine/threonine kinase (*BRAF*); cervical cancer (CC); colorectal cancer (CRC); complete response (CR); duration of response (DOR); endometrial cancer (EC); high-grade glioma (HGG); human epidermal growth factor receptor 2 (HER2); immunohistochemistry (IHC) [scoring system]; infantile fibrosarcoma (IFS); intravenous (IV); low-grade glioma (LGG); mammary analogue secretory carcinoma (MASC); median duration of response (mDOR); mitogen-activated protein kinase (*MEK*); neurotrophic tyrosine receptor kinase (*NTRK*); non-small cell lung cancer (NSCLC); objective response rate (ORR); ovarian cancer (OC); partial response (PR); per os (by mouth) (PO); rearranged during transfection (*RET*); salivary gland tumor (SGT); soft tissue sarcoma (STS); thyroid cancer (TC); treatment-related adverse effects (TRAEs). * denotes there’s no recommended dose established for patients < 26 kg.
